# Sticky Decisions: Peanut Butter in a Time of *Salmonella*

**DOI:** 10.3201/eid1605.AD1605

**Published:** 2010-05

**Authors:** Gülbanu Kaptan, Baruch Fischhoff

**Affiliations:** Carnegie Mellon University, Pittsburgh, Pennsylvania, USA (G. Kaptan, B. Fischhoff); Wageningen University, Wageningen, the Netherlands (G. Kaptan)

**Keywords:** Salmonella, peanut butter, contamination, recall, US FDA, risk perception, risk communication, bacteria, another dimension

## Abstract

We present a consumer-focused perspective on creating communications regarding potentially contaminated foods. It is illustrated with decisions that might have faced US consumers during the 2009 recalls of peanut and pistachio products. The example shows how knowledge about test results and regulatory processes might be made more useful to consumers.

## December 12, 2008

A prudent, informed consumer is about to open a jar of peanut butter. It is one of her favorite foods. Mostly, she loves the taste, but she also knows it as a cheap, healthy food—although she is a little fuzzy on those details (*1*–*3*). Like most Americans, she always has peanut butter at home. Unlike most, though, she always considers *Salmonella* risk, before opening a new jar, then decides whether to eat it, toss it, or wait a month to see if any problems turn up. She has done some research, too. Here is her reasoning.

If she eats the peanut butter and gets salmonellosis, then she has to pay for treatment and lose work time. The US Department of Agriculture (USDA) estimated that an average case in 2007 cost $1,821 in lost wages and medical costs (*4*). She can imagine her case costing less (if her insurance covers the medical costs and she uses otherwise “wasted” sick-leave days) or more (if the opposite is true), but she decides to use $1,821 in her decision making.

If she throws out the peanut butter, she will lose its $3 cost. Getting a refund is such a hassle that it will still feel like losing $3.

If she waits a month, she will incur the opportunity cost of the money tied up in the peanut butter. She puts that at $0.02 (using 8% annual percentage rate).

Now, she just needs to know the probability of salmonellosis. If it is greater than 1/607, then she should toss the jar, comparing its cost ($3) with that of getting sick ($1,821). If it is greater than 1/91,050, then she should wait a month, comparing its cost ($0.02) with that for getting sick ($1,821), assuming that food inspectors find any problem by then, making the risk zero.

But money isn’t everything.

If she throws out the jar, getting another will be a small hassle. She decides that the $3 covers that. She would feel bad about wasting the food but also feel good about her prudence. So, those psychological effects balance out. She vaguely worries that the same logic will lead her to throw away the next jar (and the next). That *would* make her feel bad.

If she waits a month, then the peanut butter might lose taste or nutritional value, or somehow “go bad.” However, she can’t find any good information about those possibilities and decides to ignore them. It is just a month.

Therefore, if she eats from the jar, the only important nonmonetary consequence is her getting salmonellosis. She knows that it usually involves an illness of 4–7 days, with diarrhea, fever, and abdominal cramps, and that most persons recover without treatment. However, the diarrhea sometimes calls for hospitalization, and the infection can spread to the blood. People can die, if not treated promptly with antimicrobial drugs (*5*). Even if USDA has not put a dollar value on suffering, she would pay a lot to avoid it. She would pay even more if she had, or was making the decision for someone with, a weak immune system.

If she tosses the jar, then she will face the risk of driving to the store to get it. She puts that at 1 chance in 100,000 of an accident, and 1 chance in 50 of that crash being fatal (*6*).

If she eats from the jar and it has any *Salmonella* bacteria, she will probably consume some of the bacteria, given how peanut butter is made. She realizes that she can put an upper bound on that risk: in 2007, a total of 1 in 6,702 Americans contracted foodborne salmonellosis, from all sources (*7*). Given that most Americans eat peanut butter, her chances must be smaller—unless there are problems.

That probability is much less than the 1/607 threshold. Therefore, based on purely economic consideration, there is no point in tossing the jar, even if she considers the suffering that USDA ignored. That probability is higher than the 1/91,050 threshold for waiting. But 1/6,702 is such a conservative estimate and there are so many nonmonetary reasons not to wait—and the peanut butter looks so good.

She knows how hard the US Food and Drug Administration (FDA) works on food safety, and she checks its website for recall notices (*8*). No reported problems! So, she opens the jar and enjoys the great taste of peanut butter.

## January 12, 2009

A month later, she buys a new jar. The next day, her morning web check finds that FDA, the Centers for Disease Control and Prevention, USDA, and others are investigating a multistate outbreak of *Salmonella enterica* serovar Typhimurium, with peanut butter the likely source. FDA is inspecting an unnamed manufacturer and tracing its distribution channels.

Looking at her new jar, she realizes that only one thing has changed since her last decision: the probability of contamination. It must be larger, but by how much? The announcement says nothing about that probability and provides no advice. She wonders what that means. Is the outbreak under control? Are they waiting for authoritative information? Is it up to the firm to issue a recall?

Without a clear signal, she opens the jar. The peanut butter tastes as good as ever, but she does not enjoy it as much. In fact, she is so troubled about what she has just eaten that she expands her online search beyond her daily visit to the recall website. She realizes that she can’t undo her exposure. However, perhaps she can get some reassurance—or faster medical attention, if need be.

The recall website mentions no product names. However, her Google search (on “peanut butter,” “Salmonella,” and “multistate outbreak”) shows that, 2 days earlier, King Nut Company voluntarily recalled peanut butter manufactured by Peanut Corporation of America (PCA) and distributed under its King Nut and Parnell’s Pride labels. King Nut says that these brands are only sold wholesale and that all its other products are safe. Although she eats another brand, she keeps worrying.

How confident can King Nut be about its other brands? Are none of the peanuts grown in the same fields, shipped in the same trucks, processed at the same facilities, or handled by the same employees? Are other companies doing their own inspections? Can FDA require tests and recalls? How good, and fast, are the tests? Without answers, what has been found does not tell her what might be found.

Over the next month, she has good news: she does not get sick. She also has bad news: seeing the outbreak explode, on her daily website checks. Although she is fine physically, she feels like she has dodged a bullet. She cannot understand the 2-day lag between the announcements by the government and by King Nut, which she kicks herself for having missed. Had she gotten sick, would she have found the information that she needed to get timely treatment?

## February 12, 2009

It’s time to buy more peanut butter. However, a lot has happened during that month. On January 13, PCA announced a voluntary recall of 21 lots of peanut butter and peanut paste, produced in its Blakely, Georgia, USA, facility. By January 27, it had expanded the recall 3 more times. The next day, it recalled all dry- and oil-roasted peanuts and peanut products processed at Blakely since January 1, 2007. On January 30, FDA confirmed reports of a criminal investigation of PCA, for continuing to ship products after receiving positive *Salmonella* tests. Hundreds of persons are sick; 9 die. The case-fatality rate is about that of previous outbreaks, which suggests that the strain is not unusual, although the scope is.

The recall website now provides consumer recommendations and a searchable database, for recalled products. Her favorite brand is not on the list, so she still believes that her risk is negligible. She makes her usual monthly purchase, then has a moment of truth, when she gets home: she still does not know how anyone decides which products to test or what information to share. That 2-day lag still bothers her, as does wondering when the criminal investigation began. She is unhappy about her “no news is good news” inference, last month, and maybe this one, too.

She knows that FDA’s employees work hard. She is devoted to its websites. However, she also knows that there are limits to its resources and legal authority. She just doesn’t know what they are. So, she makes some guesses.

Given the stream of new recalls, she concludes that FDA waits for strong positive evidence before saying anything. As a result, she can’t tell whether her favorite brand has been cleared or just not yet tested. Her rule is still to toss a jar if the risk is over 1/607. The recall list currently has ≈50 peanut products. There would have to be over 30,350 (50 × 607) peanut products, for the rate to be under her threshold. She should toss the jar, if there is a similar rate among products that have not been officially cleared.

But what does she know? Maybe she should be worried that other foods are processed, shipped, or shelved along with the peanut butter. She knows that life has risks and she is willing to take reasonable ones. But she hates not knowing what’s going on.

In the following days, the number of *Salmonella* cases increases, confirming her fears. Still, there is no recall for the major national brands, including her own. That’s good. She just doesn’t know how good, without understanding what gets tested and announced.

At least she can eat her second favorite nut product, pistachios.

## March 12, 2009

Her month of watchful waiting has passed. Her morning website checks have found a continuing but slackening stream of recall notices and salmonellosis cases. FDA reports conducting more audits and inspections and collaborating with other authorities. It still has found no contamination in major national brands. Although that message has not changed, she assumes that the supporting evidence is now stronger. Still, she is unnerved enough not to open her jar or to buy other peanut products, just in case she has missed something. She’ll stick with pistachios.

During the month, she had a disquieting experience: She returned a box of peanut butter granola bars. The merchant refunded her money, no questions asked. However, she found that the product was still on the shelves, while her favorite granola bar, with the tiny chocolate chips, was missing. She guesses that the store was humoring her, by giving a refund for a safe product, while peanut butter was a micro ingredient in the chocolate chip bar. The refund gets her wondering whether food manufacturers are more careful with products featuring peanut butter, compared to ones where it is a trace ingredient (or an “industrial chemical”). She hopes that qualified people worry about these things. She just wants useful information.

## March 31, 2009

Her morning web check reveals the shocking news that the second-largest US pistachio processor (Setton) has voluntarily recalled certain lots of roasted nuts. She never worried about pistachios before. However, if they can be contaminated, then she faces the same decision as with the peanut butter. The cost is even the same. To control her passion for pistachios, she buys small ($3) packages.

She has a harder time figuring out the risks. The announcement advises that “Consumers should not eat pistachios or food products containing them until they can determine that the products do not contain pistachios recalled by Setton.” How can she tell? Are all those products sold under the Setton name? What does it mean that untested pistachios are suspect, whereas only tested peanuts were? Are the risks that different? Perhaps pistachio and peanuts are processed differently. Perhaps Setton is less trustworthy than PCA (criminal investigation notwithstanding). Perhaps the authorities know more than they are allowed to reveal. Perhaps the reporting policy has been changed. If so, how? Are they being hypercautious? Can she then be totally confident about almonds, her third favorite nut? Or, are they being so cautious that everything will soon be suspect? If so, perhaps she should just eat those pistachios.

## April 12, 2009

A month has passed without her favorite brand appearing on the recall list. She is about to open the jar, when her morning Internet check finds that a company named Westco/Westcott has “declined” FDA’s request to recall its products with PCA peanuts and “to provide access to certain records about the distribution of these products.” In response, FDA has asked US Marshals to execute an inspection warrant (*9*). She is shaken to learn that FDA cannot require food recalls. Realizing how little she knows about FDA’s authority and resources, she decides to let her poor jar sit for another month.

## What Does She Want?

One morning, she notices a “Contact Us” option at the recall website. She thinks, “What do I want? I know that they’re working hard to protect me. But, somehow, I’m not quite getting the information that I need to protect myself. I like the notices’ standard format. I know how to find things on it. I’ve learned to decode most of the jargon. I just don’t know what it all means, in terms of my risks. Maybe if I grade some of the postings, it will clarify my thinking”:

January 12 (*10*). Grade: D. Although I learned that there was an outbreak, possibly related to peanut butter, I did not learn anything about what to do, even though the King Nut recall was already happening. Seeing how complex the peanut production system is, I feel like they must have suspected that they had not found all the problems. They could have said, “We recommend not eating peanut products until we do more testing. Don’t throw them away, though; they may be fine.” They could also have said that they cannot force recalls or do all the testing that they want, so that I would know what they are up against.

January 16 (*11*). Grade: B. By saying that they could not say which brands to avoid, they allowed me to make a better decision. Still, it was unnerving to see such a big change, over 4 days, without an explanation why.

January 17–19 (*12*–*14*). Grade: B+. They recommended the decision that I would have made on my own, had I known what they knew. Still, I was left wondering about the PCA controversy and what it meant about future surprises. It would have helped just to hear, “We cannot comment on the ongoing criminal investigation of PCA.”

After thinking through these dates, she knows what she wants and thinks that it wouldn’t be too hard to do:

- *Talk to consumers*. Find what decisions we face and what we worry about. I am cooking for one person and can afford to wait. Other people can’t.
- *Tell us what you know—and don’t know*. We would like you to be certain but don’t want to learn too late about possible problems.
- *Tell us if your hands are tied*. If we expect you to do the impossible, then we’ll get mad at you, not at those who keep you from protecting us.
- *Get the information that we need*. Do the tests that will tell us what not to eat. We don’t care that much about “all clears.”
- *Have some ordinary people read each message before you post it*. If they understand it, then most everyone will.

Some of what she wants is happening already. For example, in January 2010, FDA established a new website, FDA Basics, to inform consumers about how FDA works (*31*).

## Coda

Our hypothetical consumer is unusually, but not implausibly, thoughtful about her food safety decisions (and her love of peanut butter), given the picture that emerges from the large research literature on the topic (*15*,*16*). Although consumers’ safety behavior is often disappointing, some of those failures reflect their difficulty understanding what to do (*17*–*23*). Risk communication research often finds large gaps between what experts say and what consumers hear—and need to hear. Fortunately, research can close much of that gap, allowing public health officials to do all that is possible to help people to make wise choices in an uncertain world (*24*–*31*).
